# NEK1-Mediated Phosphorylation of YAP1 Is Key to Prostate Cancer Progression

**DOI:** 10.3390/biomedicines11030734

**Published:** 2023-02-28

**Authors:** Ishita Ghosh, Md Imtiaz Khalil, Rusella Mirza, Judy King, Damilola Olatunde, Arrigo De Benedetti

**Affiliations:** 1 Department of Biochemistry and Molecular Biology, Louisiana State University Health Shreveport, Shreveport, LA 71103, USA; 2 Department of Pathology, Louisiana State University Health Shreveport, Shreveport, LA 71103, USA

**Keywords:** TLK, NEK1, YAP1, prostate cancer, AR, EMT

## Abstract

The key to preventing mCRPC progression is understanding how androgen-dependent PCa cells progress to independence and modify their transcriptional repertoire accordingly. We recently identified a novel axis of the Hippo pathway characterized by the sequential kinase cascade induced by androgen deprivation, AR^−^>TLK1B>NEK1>pYAP1-Y407, leading to CRPC adaptation. Phosphorylation of YAP1-Y407 increases upon ADT or induction of DNA damage, correlated with the known increase in NEK1 expression/activity, and this is suppressed in the Y407F mutant. Dominant expression of YAP1-Y407F in Hek293 cells reprograms the YAP1-mediated transcriptome to reduce TEAD- and p73-regulated gene expression and mediates sensitivity to MMC. NEK1 haploinsufficient TRAMP mice display reduced YAP1 expression and, if castrated, fail to progress to overt prostate carcinomas, even while displaying reduced E-Cadherin (E-Cad) expression in hyperplastic ductules. YAP1 overexpression, but not the Y407F mutant, transforms LNCaP cells to androgen-independent growth with a mesenchymal morphology. Immunohistochemical examination of prostate cancer biopsies revealed that the pYAP1-Y407 nuclear signal is low in samples of low-grade cancer but elevated in high GS specimens. We also found that J54, a pharmacological inhibitor of the TLK1>NEK1>YAP1 nexus leading to degradation of YAP1, can suppress the transcriptional reprogramming of LNCaP cells to androgen-independent growth and EMT progression, even when YAP1-WT is overexpressed.

## 1. Introduction

Prostate cancer (PCa) is the second leading cause of cancer death in men in the Western world. The disease progression has been defined by characteristic features correlated with its aggressiveness/prognosis: from Prostate Intraepithelial neoplasia (PIN) to prostate adenocarcinoma (PRAD), to castration refractory PCa (CRPC), to neuroendocrine PCa (NEPC), both in patients from engineered mouse models and clinical studies. The mechanism of the disease advancement and treatment modalities are still under investigation.

The Hippo pathway, the evolutionarily conserved developmental pathway known to regulate organ size, proliferation, apoptosis, cell migration, stemness, etc. [1,2,3]. has also been implicated in PCa tumorigenesis [4]. YAP/TAZ (60% identical) are the main effectors of the Hippo signaling pathway, which is involved in regulating organ size through controlling multiple cellular functions, including cell proliferation and apoptosis. The Hippo pathway responds to a variety of signals, including cell–cell contact, mechanotransduction, and apico-basal polarity [1,2,3]. When the Hippo pathway is activated, kinases MST1/2 and LATS1/2 phosphorylate and inactivate YAP and TAZ. YAP and TAZ are transcriptional co-activators but lack DNA binding activity. Upon phosphorylation by MST and LATS kinases, they are sequestered in the cytoplasm, ubiquitylated by the β-TrCP ubiquitin ligase, and marked for degradation by the proteasome. YAP/TAZ are usually inhibited by the cell–cell contact in normal tissues [2].

Over-activation of YAP/TAZ through aberrant regulation of Hippo has been noted in many types of tumors and associated with the acquisition of malignant traits, including resistance to anticancer therapies, maintenance of cancer stem cells, distant metastases and in prostates and Androgen Independent (AI) adenocarcinoma progression [5,6]. When the Hippo core kinases are “off,” YAP/TAZ translocate into the nucleus, bind to TEAD1-4, and activate the transcription of downstream target genes. This leads to multiple oncogenic activities, including loss of contact inhibition, cell proliferation, epithelial–mesenchymal transition, and resistance to apoptosis. In PCa, YAP1 has been identified as a binding partner of Androgen Receptor (AR) and co-localized with AR in an androgen-dependent manner and in an androgen-independent (AI) manner in Castrate Resistant Prostate Cancer (CRPC) [5] YAP was also found to be upregulated in LNCaP-C42 cells and, when expressed ectopically in LNCaP, activated AR signaling and conferred castration resistance, motility, and invasion (reviewed in [4]). Knockdown of YAP greatly reduced the rates of migration and invasion of LNCaP, and YAP activated AR signaling was sufficient to promote LNCaP cells from an Androgen Sensitive (AS) state to an AI in vitro, and castration resistance in vivo [7,8]. It was also recently determined that ERG (and the common *TMPRSS2-ERG* rearrangement) activates the transcriptional program regulated by YAP1, and that prostate-specific activation of either ERG or YAP1 in mice induces similar transcriptional changes and results in age-related prostate tumors. Transcriptional co-activator YAP1, which acts downstream of the canonical Hippo kinases [1], is known to interact with a plethora of proteins, including TMPRSS2-ERG and the AR in PCa (rev. in [4]). However, how non-canonical Hippo kinases can regulate YAP1 in this disease has not yet been determined.

We previously found that NIMA-related kinase 1 (NEK1) interacts with YAP1 and phosphorylates it at six sites, including Y407, which was identified earlier in phospho-proteomic studies but not investigated [9]. Here we report that the phosphorylation of Y407 is key for YAP1 stability and transcriptional co-activation function. The Y407 resides in the transactivation domain (TAD), and this residue is equivalent to pY316 of TAZ, which was shown that when phosphorylated, reportedly by c-Abl, was necessary to mediate its interaction with the transcription factor NFAT5 [10]. Our interest in studying the consequence of Y407 phosphorylation was initiated following previous studies that reported that the phosphodegron domain (376–396) is located in proximity to Y407 [3]. Phosphorylation close to this region may affect the stability of the protein, and we previously showed that overexpression of a NEK1-T141A dominant negative mutant in LNCaP cells results in the enhanced degradation of the YAP1 protein [9]. In addition, this region belongs to the TAD and may therefore regulate its overall coactivator activity [11]. Importantly, only NEK1 (beside NEK10 which is not elevated in cancer) is a dual-specificity kinase [12]. Therefore, inhibition of NEK1 activity cannot be compensated by any other Nek family member for pY407-mediated stabilization. 

We previously reported that androgen deprivation therapy (ADT) or anti-androgens results in the activation of the TLK1B>NEK1>YAP1 pathway and induction of EMT and stemness genes, which we proposed to be critical for mCRPC progression and correlated with bioinformatics analyses of PCa data in TCGA [9]. Identification of molecular markers for PCa progression and treatment response is one of the most important areas of investigation. One key challenge lies in identifying the molecular pathways that cause a shift from an androgen-dependent to androgen-independent state of growth in these cancer cells. We now provide evidence that the NEK1>YAP1 activation pathway is critical for PCa development and CRPC progression in the TRAMP model and in LNCaP cells, confirming previous work which showed that YAP1 overexpression in LNCaP cells rapidly converts them to androgen-independent (AI) growth [4]. We further present evidence that targeting the activity of TLK1 as a druggable upstream activator of the NEK1>YAP1>CRPC pathway can be an important step in the management of PCa along with standard anti-androgen therapy in combination with a powerful inhibitor of TLK1 such as J54 [13] (rev. in [14]).

## 2. Materials and Methods

### 2.1. Plasmids and Antibodies

pFLAG-YAP1 was a gift from Yutaka Hata (addgene plasmid # 66853) and pEGFP-C3-hYAP1 was a gift from Marius Sudol (addgene plasmid # 17843), addgene (Watertown, MA, USA). The following antibodies were used in this study: mouse anti-YAP (dilution- 1:1000 in 5% Milk+TBST, Santa Cruz Biotechnology, SCBT, cat# sc101199, Dallas, TX, USA), rabbit anti-phospho-YAP-Y407 (custom generated by Life Technologies, Carlsbad, CA, USA), mouse anti-NEK1 (1:1000 in TBST, SCBT, cat# sc-398813, Dallas, TX, USA), rabbit anti-phospho-NEK1 pT141 (lab-generated by Life Technologies, Carlsbad, CA, USA), HRP-conjugated anti-β-tubulin (1:1000 in TBST, SCBT, cat# sc-23949, Dallas, TX, USA,), anti-FLAG (1:1500 in 5% Milk+TBST, Cell Signaling Technology, CST, cat# 14793S, D6W5B, Danvers, MA, USA), anti-GAPDH (1:1300 in 5% BSA+TBST, CST, cat# 2118S (14C10), anti-GFP (Thermo Fisher, cat# MA5-15256 (GF28R), Waltham, MA, USA), anti-phospho-ATR (T1989) (1:1000 in 5% Milk+TBST, CST, cat# 58014S), anti-BAX (1:1000 in TBST, SCBT, cat# sc-23959 (6A7),), E-Cad (1:1000 in TBST, CST, cat# 3195S,), N-Cad (CST, cat# 13116S), and rabbit anti-actin (1:1000 in TBST, Abcam, cat# ab1801, Cambridge, MA, USA). Secondary HRP-conjugated antibodies, anti-rabbit (CST, cat# 7074S) and anti-mouse (CST, cat# 7076S) were used to probe immunoblots.

### 2.2. Cell Treatment

LNCaP GFP, LNCaP GFP-YAP1-WT, and LNCaP GFP-YAP1-Y407 were grown to 70–80% confluency in RPMI media. Cells were then treated with 10 µM J54 for 24 h. Thereafter, the treated cells were harvested for RNA extraction. For mitomycin C (Sigma-Aldrich, cat# M-0503, St. Louis, MO, USA) treatment, Hek293 cells were treated with 3 μM MMC for 24 h or as indicated. Treated cells were collected and processed for cell lysate for RNA extraction or immunoblotting. For the cycloheximide chase assay, 50 μg/mL of CHX (Sigma-Aldrich, cat# C-7698, St. Louis, MO, USA) was used as the final concentration. Cells were collected at indicated times and processed with a RIPA buffer for immunoblotting. The cell viability assay was performed using MTT as per the manufacturer’s instruction (Promega Corp., CellTiter 96^®^ Aqueous reagent, cat# G3580, WI, USA).

### 2.3. RNA Extraction

RNA extraction used 2.5 × 10^6^ cells. The cells were homogenized in 0.35 mL of trizol. The cell lysate was homogenized and centrifuged at 12,000× *g* for 5 min (4 °C) to obtain the supernatant. A total of 90 µL of chloroform was added and the reaction mixture was centrifuged for 15 min at 12,000× *g* (4 °C). A total of 180 µL of propanol was added and incubation was carried out under the same condition. The pellet obtained was resuspended in 75% ethanol and centrifuged at 7500× g for 10 min (4 °C). The pellet was resuspended in RNAse-free water. Concentration and yield were obtained thereafter.

### 2.4. Realtime Quantitative PCR (RT-qPCR) for p73, TEAD Targets, AR and EMT Genes

Following the manufacturer’s instructions, total RNA was further purified using a Qiagen RNeasy RNA isolation minikit (catalog number 74,104, Germantown, MD, USA). By using the ProtoScript II First Strand cDNA synthesis kit and 1 µg of total RNA per reaction, complementary DNA (cDNA) was synthesized (New England Biolab, cat# E6560S, Ipswich, Massachusetts, USA). iQ SYBR green supermix (Biorad, cat# 1708880, Des Plaines, IL, USA) and Bio-Rad CFX96 Fast Real-Time PCR Systems were used to perform qPCR.100ng of cDNA was used per RT-PCR reaction. The ΔΔCt relative quantification method was used to determine changes in gene expression. Actin mRNA was used as an internal control. Each value is represented by its mean ± standard error (SEM).

### 2.5. Scratch-Wound Repair Assay

Mouse parental NT1 PCa cells and two Nek1-KO NT1 clones were previously described and assayed for the expression of several YAP/TEAD-dependent Epithelial-Mesenchymal Transition (EMT) markers [9] They were tested for motility using the scratch wound repair assay with the Incucyte system, as previously described [15].

### 2.6. Immunohistochemistry

For human tissues, representative tissues of prostatic adenocarcinoma were selected by our pathologist from a few radical prostatectomy specimens. Tissues were fixed with 10% formalin, paraffin embedded (FFPE). TRAMP mouse and human prostate tissue cut in 5 µm and 4 µm thin sections, respectively, were used for this study. De-paraffinization was performed in xylene, followed by rehydration in decreasing alcohol concentrations as follows: 100%, 90%, 80%, 70%, and 50% ethanol. Antigen unmasking was performed using a sodium-citrate–EDTA buffer (10mM Na-Citrate (pH-6.02) and 1mM EDTA) in a microwave at a 30% power setting for 10 min. After slide cooling, endogenous peroxidase was blocked in 3% H2O2 in methanol for 15 min. After PBS washes, a blocking buffer was used (1.5% horse serum in PBS) for 30 min at room temperature in a humidified box. Primary antibody was diluted in 1% horse serum in PBS at the following dilutions: for YAP1- 1:50; for pYAP1-Y407- 1:50; for E-Cadherin (E-Cad)- 1:400; for pNek1- 1:50; and for N-Cad- 1:200. IHC staining of N-cadherin protein of the prostate tissue sections was conducted as previously described [15]. Incubation with the primary antibody was performed overnight at 4 °C. Slides were washed three time in PBS. Biotinylated secondary antibody (anti-rabbit/mouse) from Vectorlabs (Vector Laboratories, cat# PK-6200, Burlingame, CA) was used for 1 h incubation at room temperature. Following secondary antibody incubation, the ABC reagent from Vector (Vector Laboratories, Burlingame, CA, cat# PK-6200) was added to amplify the signal for 1 h. DAB substrate (Vector Laboratories, cat# SK-4105, Burlingame, CA, USA) staining was performed for 2–10 min each section, depending on the target antigen. FFPE sections were counterstained with standard hematoxylin and eosin staining protocol. Slides were dehydrated using a gradation of alcohol 50% and 70% ethanol followed by xylene. Slides were mounted using gelatin–glycerol mounting media and imaged. Image acquisition was completed in a bright-field Olympus microscope (Model: BX 43, Center Valley, PA, USA).

### 2.7. Animal Maintenance and Procedures

TRAMP-*NEK1^+/+^* or TRAMP-*NEK1^+/^*^−^ mice were maintained at LSU Health Science Center-Shreveport’s animal facility. Mice were castrated at 16–18 weeks of age under anesthesia and euthanized 4 weeks post-castration. All the animal experiments were approved by the Institutional Animal Care and Use Committee and by the DoD-ACURO review board.

### 2.8. Ethics Approval and Consent to Participate

The human patient samples were collected after the written consent of each subject. The identity of each donor was anonymous, and only minimal clinical information was available to the investigators. The study methodologies conformed to the standards set by the Declaration of Helsinki and the entire study was approved by our IRB. 

### 2.9. Statistical Analysis

Statistical analyses were performed using Graphpad Prism 9 and Microsoft Excel software. Data quantifications are expressed as mean ± standard error of the mean (SEM). Statistical significance was calculated by a 2-tailed Student’s *t*-test when comparing the mean between two groups, or by one-way or two-way ANOVA, followed by Tukey’s post hoc analysis when comparing more than two groups. Any *p*-values  <  0.05 were considered significant.

## 3. Results

### 3.1. NEK1 Phosphorylates YAP1 at Y407 and Increases Its Stability

**Figure 1:**
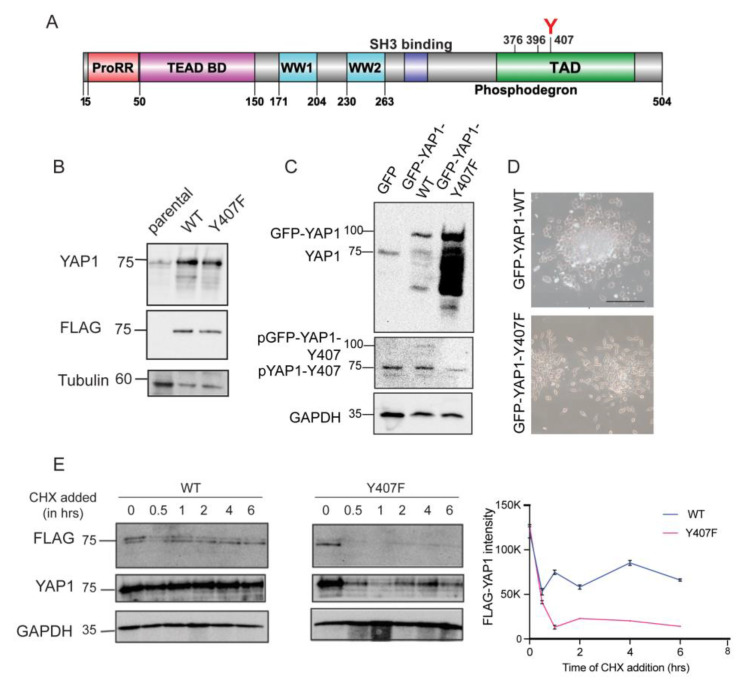
Expression of YAP1-WT and Y407F mutant in Hek293 and LNCaP cells. (**A**) YAP1 domain map showing full-length human YAP1 (504 aa) with different regions. Proline-rich region (ProRR), TEAD binding domain (TEAD BD), WW domains (WW1 and WW2), SH3 binding region, phosphodegron motif (376–396 aa) within the transcription activation domain (TAD) in proximity to Y407. (**B**) Immunoblot showing the expression of the FLAG-tagged YAP1-WT and Y407F mutant, and endogenous YAP (parental) in Hek293 cells examined with pan-YAP or FLAG antiserum. (**C**) Expression of GFP-YAP1-WT and Y407F mutant in LNCaP cells was probed with the anti-YAP1, anti-GFP, and anti pYAP1-Y407 antibody. (**D**) Specific morphology alteration of LNCaP cells expressing GFP-YAP1-WT. Note the rounded appearance and overlapping growth pattern, which was not seen with the Y407F mutant-expressing cells that retained “normal” LNCaP morphology. Scale bar is 100 μm. (**E**) Cycloheximide chase assay (CHX) performed at indicated timepoints to determine the stability of the YAP1-WT and YAP1-Y407F protein in Hek293 cells. Immunoblots probed with indicated antibodies. Quantification of FLAG-YAP1 levels from CHX chase is shown to right. Results from three independent experiments ± SEM plotted.

Previous studies showed that NEK1 overexpression leads to accumulation of YAP1, whereas expression of the NEK1-T141A hypoactive mutant displays enhanced YAP1 degradation [9]. In this study, we report the consequences of NEK1-dependent YAP1 stabilization. We confirm that NEK1 phosphorylates YAP1 on Y407 site, which lies in the transcription activation domain of YAP1 that also contains a degron motif (Figure 1A). We hypothesized that the Y407 phosphorylation could increase the interaction of YAP1 with its transcriptional partners and hence lead to its stabilization and to stronger transcriptional activity. Phosphorylation of YAP1 homolog TAZ on Y316 has been implicated in regulating NFAT5 transcriptional activity under hyperosmotic stress. Y316 on TAZ is held equivalent to Y407 on YAP1. 

We found that NEK1 is the first and perhaps only kinase that phosphorylates YAP1 on Y407, so we generated site-directed mutation on human YAP1 cDNA encoding plasmid at Y407 to phenylalanine which cannot be phosphorylated. We transfected Hek293A cells with YAP1-WT and YAP1-Y407F variant plasmids and generated stable cell lines expressing YAP1-WT and YAP1-Y407F (Figure 1B). We hypothesized that NEK1-mediated phosphorylation of YAP1 at Y407 would stabilize the protein, so we tested this with a cycloheximide chase assay (Figure 1E).

We found that YAP1-WT was stable for at least 6 h, while Y407F was degraded even at the earliest 30 min time point (Figure 1E, WB shown for the FLAG and total YAP1 and relative levels of FLAG-YAP1 quantified, Figure 1E). This implicated that the phosphorylation of YAP1 by NEK1 was important for the stability of the protein. Note that the FLAG-YAP1 protein began to accumulate slightly at later times because CHX was used at only 50 µg/mL concentration to avoid potentially complicating toxic effects, which did not completely inhibit protein synthesis.

In order to confirm the generality of the Y407F instability and the importance of NEK1-mediated YAP1 phosphorylation, we stably transfected LNCaP PCa cells with GFP-YAP1-WT and GFP-YAP1-Y407F and examined YAP1 expression. LNCaP expressing only GFP was used as the control, and the results are shown in Figure 1D,E. The expression of the ~75 kDa endogenous YAP1 was clearly seen in the LNCaP-GFP cells, whereas the ~100 kDa GFP-YAP1 band was seen in the GFP-YAP1-WT and Y407F-expressing cells.

Interestingly, the cells expressing the GFP-YAP1-Y407F protein, apart from the full size 100 kDa protein, showed an extensive pattern of smaller, partially degraded products. This can be explained by our observation of the high instability of the YAP1-Y407F mutant (in Figure 1E), and is consistent with our previous observation that the hypoactive NEK1-T141A-expressing LNCaP cells display high levels of YAP1 cleaved/degraded products [9]. In the same line, we noticed that the GFP-YAP1-Y407F cells also expressed 2–3 times more full-length protein than the WT counterpart (Figure 1C), possibly to compensate for its high instability, as this may confer some advantage to these cells.

**Figure 2:**
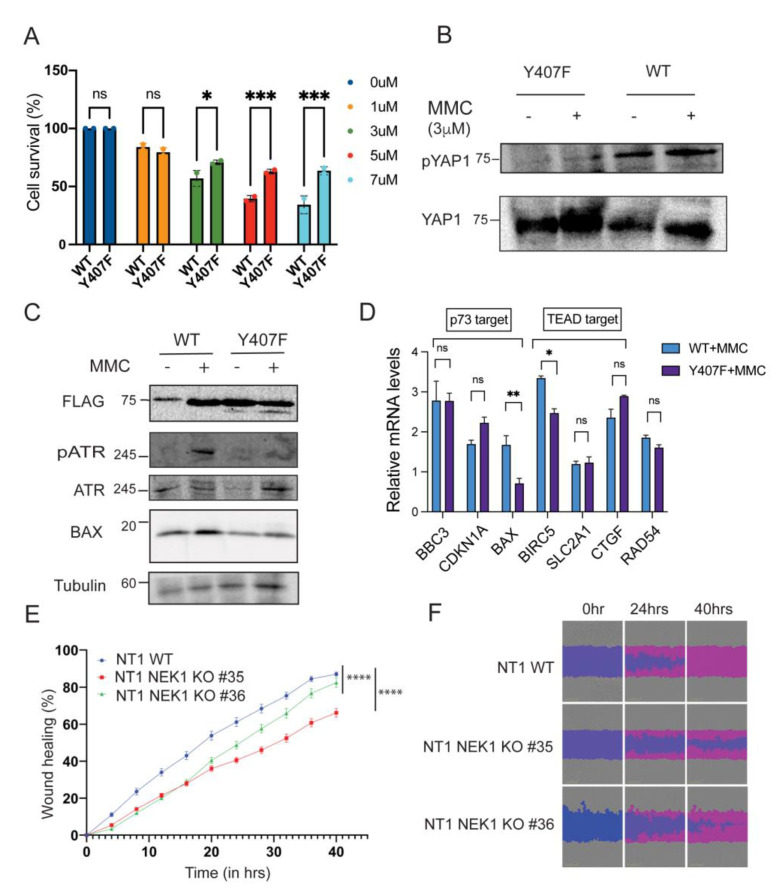
Exogenous YAP1 expression (WT and Y407F mutant) resulted in the altered expression of pro-apoptotic genes following MMC treatment, while NEK1-KO cells displayed reduced motility. (**A**) Survival analysis of Hek293 expressing FLAG-YAP1-WT or the Y407F mutant exposed to different concentrations of MMC for 7 days. The sensitivity of parental Hek293 cells to MMC was previously reported [17] and is similar to the sensitivity profile of cells expressing FLAG-YAP1-WT. (**B**) pYAP1 (pYAP1-Y407) level increased post MMC induction in YAP1-WT overexpressing cells. The phosphorylation of YAP1-Y407 was confirmed with a phospho site-specific (Tyr 407) antibody, which confirmed that its signal increased following treatment with MMC, which activated NEK1. This led to increased stability and the accumulation of YAP1, endogenous and FLAG-tagged combined. Note that the Y407F mutant did not show a pYAP1 signal or protein accumulation after MMC. (**C**) Expression of FLAG-YAP1-WT was elevated after MMC treatment for 24 h, likely via NEK1-activated stabilization; this was not observed with the Y407F mutant. Evidence that MMC activates appropriate DDR was confirmed by the presence of pATR-T1989 (and a slight increase in total ATR). Increased BAX expression demonstrated activation of the pro-apoptotic response to MMC and was reduced in the Y407F-expressing cells compared to WT. (**D**) Comparison of the expression of typical YAP1/TEAD (pro-tumorigenic genes) and YAP1/p73 (pro-apoptotic genes) in Hek293 cells after 24 h of MMC induction. Relative mRNA levels for p73 target genes—BBC3 (PUMA), CDKN1A (p21) and BAX analyzed and TEAD target genes—BIRC5 (survivin), SLC2A1 (GLUT1) and CTGF analyzed from three independent experiments. Two-way ANOVA statistical test performed with comparison of each cell mean with the other cell mean in that row. (* *p* < 0.05; ** *p* < 0.01; *** *p* < 0.001) (**E**). (**F**) Scratch-wound repair assay performed on NT1 (mouse PCa cells) or NT1-NEK1 KO cells in two clones to determine the 2D migration rate by plotting relative wound density against different time points. One-way ANOVA followed by Tukey’s post hoc analysis was used for multiple group comparison (**** *p* < 0.0001). Each data point contained 8–12 replicates. Error bar represents SEM (**E**). A representative image of wound repair shown at 0 h, 24 h and 40 h time points (**F**). These cells were previously described and shown to activate pro-motility (YAP1/TEAD-dependent) EMT genes [9].

We also generated a pYAP1-Y407 specific antiserum, and could confirm that only the YAP1-WT, and not the Y40F mutant, showed a specific signal (Figure 1C). The phosphorylation decreased when NEK1 was depleted in Hek293 cells by siRNA (Figure S1). The fact that the phosphor-signal was not completely gone could be due the presence of some residual NEK1 protein, despite the generally effective level of depletion. Moreover, the phosphorylation increased by 50% upon treatment of Hek293 cells with Mitomycin C (MMC), which causes DNA damage and activates NEK1 [16] to phosphorylate YAP1 (Figure 2B and Figure S2); however, part of the signal elevation can be explained by the concomitant stabilization and accumulation of the protein (combined endogenous and FLAG-tagged; Figure 2B).

We further assessed the pY407 antibody on the GFP-YAP1 (WT and Y407F mutant)-expressing LNCaP cells. We could confirm that the antibody detected the pYAP1-Y407 signal of the endogenous YAP1 in all three cell lines, but only weakly detected the pGFP-YAP1-WT and not the GFP-YAP1-Y407F mutant (Figure 1C, middle panel). Re-probing the blot for GFP confirmed that the pYAP1-Y407 Ab (also referred as P-Ab) detected the same GFP-YAP1 band in that position, and not the endogenous YAP1 (not shown). The low phosphorylated signal detected in the GFP-YAP1-WT could be explained by the idea that the presence of the GFP domain could hinder the accessibility of the Y407 residue to NEK1, and result in a lower signal than for the endogenous YAP1, despite the greater expression of the GFP-YAP1 proteins.

### 3.2. NEK1-Dependent Phosphorylation of YAP1 Leads to Increased YAP1 Transcriptional Activity upon Mitomycin C (MMC)-Induced DNA Damage

To test for the consequence of the phosphorylation-mediated stabilization, we asked if DNA damage would alter the survival capacity of the cells expressing Y407F, since YAP1 tyrosine phosphorylation by c-Abl was previously demonstrated to be a critical step in adaptation to the proapoptotic program in response to DNA Damage (DDR) [18,19]. To test the activity of YAP1 and the Y407F mutant in the DDR, DNA damage induction with different concentrations of MMC (1–10 μM) showed that the WT-expressing cells had a lower percent of survival, while Y407F survival was lowest at 3 μM and did not change at higher concentrations (Figure 2A). This suggests that WT protein-expressing cells had a greater apoptotic response to DNA damage induction. Note again that, considering only the FLAG and not the P-Ab (Figure 2B), the overexpressed proteins were similarly present, unless CHX was added. Thus, presumably, YAP1-WT had greater activity as a pro-apoptotic driver, likely in conjunction with p73 [18,19]. 

To examine if the pY407 modification leads to greater activity of YAP1 as a co-activator, we next tested for the expression of key genes regulated via YAP1 after the induction of DNA damage by MMC (intra-strand crosslinking agent). This results in specific YAP1/p73 mediated responses [20] and in cross-stabilization of YAP1 and p73 by preventing Itch-mediated proteasomal pathway. We confirmed that upon MMC induction, DNA damage occurred as pATR was induced (Figure 2C). FLAG-YAP1-WT expression was modestly increased with MMC (Figure 2B,C), which could be explained by the known increased expression and activity of NEK1 [16] resulting in greater YAP1 phosphorylation-mediated stabilization. In contrast, the expression of Y407F remained unaltered even with MMC, suggesting that the phosphorylation of Y407 by NEK1 is a key determinant for its stabilization/accumulation and possibly for its activity via increased interaction with p73. 

Overall, the YAP-related transcriptional changes cannot be explained by minor differences in expression between WT and mutant YAP1 proteins. Indeed, in relation to the p73 target gene, BAX expression was significantly greater in WT cells compared to Y407F post MMC induction (Figure 2C). As a control, we tested the expression of RAD54, which is known to be induced upon DNA damage [21] independently of YAP1 activity, and its expression was indeed modestly (but equally) increased in the two cell lines with MMC. Overall, this suggests that YAP1-WT has greater transcriptional co-stimulatory capacity (besides greater stability) than the unphosphorylatable Y407F.

Since YAP1 plays an important role in cell migration and motility, and we demonstrated that NEK1 phosphorylates YAP1 to regulate its transcriptional activity, in addition to testing the effect of NEK1 on the DDR [22], we wanted to assay if NEK1 can regulate cell migration capacity through the regulation of YAP1 activity. We thus performed a wound repair assay with two different clones of NEK1 KO NT1 (mouse PCa) cells with a demonstrated reduced expression of mesenchymal markers, N-Cad, Twist1, and Zeb1 [9], and our results indicate that cell migration capacity was clearly NEK1-dependent (Figure 2E,F).

### 3.3. YAP1 Overexpression, but Not the Y407F Hypoactive/Unstable Mutant, Transforms LNCaP Cells to Androgen-Independent Growth

We wanted to establish if there were growth differences between LNCaP cells expressing GFP-YAP1-WT or the Y407F mutant. This could likely reflect the fact that the Y407F mutant is unstable, so the cells must compensate by maintaining a higher output. Alternatively, both lines may have a selective advantage for growth when YAP1 is overexpressed, even assuming that the Y407F has lower transcriptional activity. 

**Figure 3:**
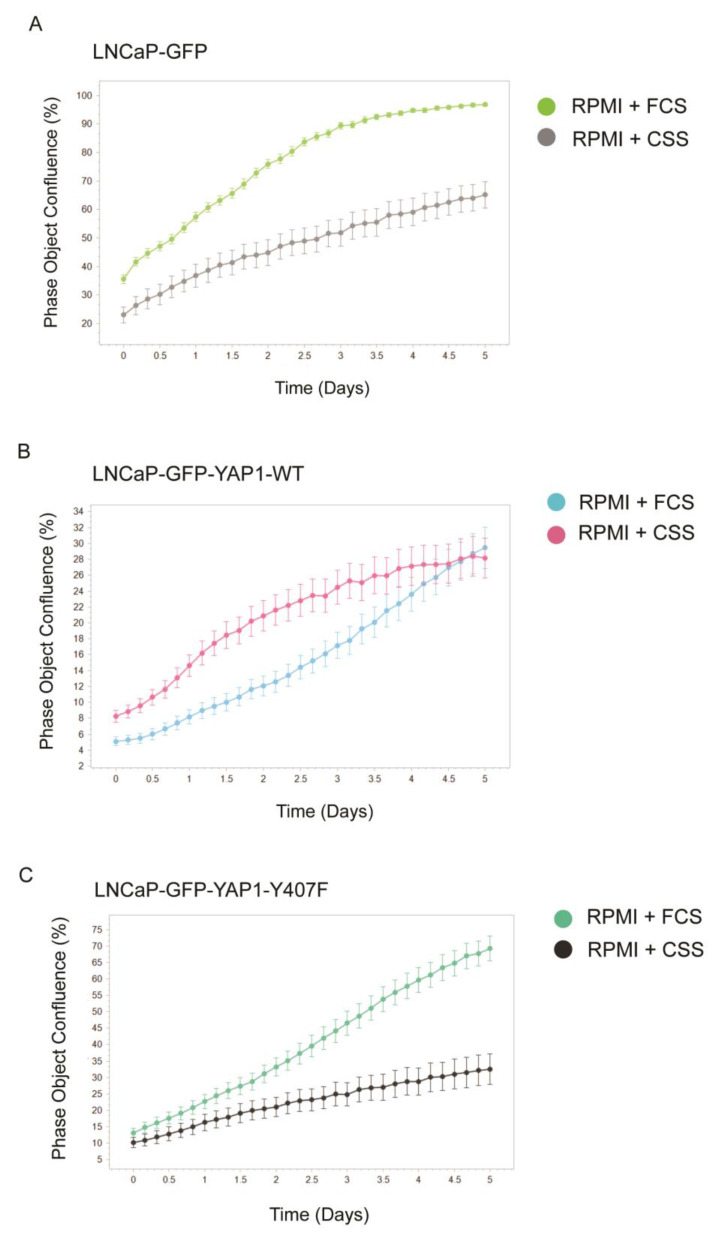
Only LNCaP cells expressing GFP-YAP1-WT were androgen-independent for growth. (**A**) LNCaP-GFP control cells exhibited slower growth in androgen-deprived media (charcoal stripped serum—CSS) compared to normal complete growth media (with Fetal calf serum, FCS). (**B**,**C**) LNCaP cells expressing GFP-YAP1-WT, but not the Y407F mutant were intrinsically androgen-independent (AI) for growth shown for indicated time. LNCaP cells expressing GFP-YAP1-WT, GFP-YAP1-Y40F, or vector control GFP, were plated in 96 well plates at 10,000 cells per well and monitored for growth over a week period with the Incucyte.

**Figure 4:**
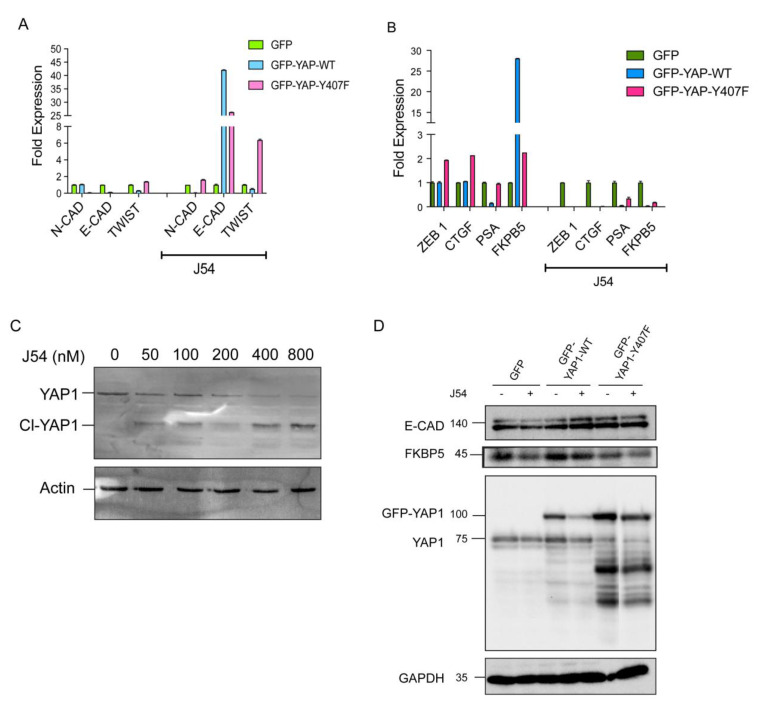
Changes in EMT and AR driving genes were reversed by J54 in LNCaP cells overexpressing GFP-YAP1. (**A**) Expression of key EMT genes in LNCaP cells overexpressing the GFP-YAP1-WT and Y407F mutant. Relative level of mRNA is shown for N-CAD, E-CAD and Twist genes treated with or without J54 (10 μM, 24h). (**B**) Same experiment as (**A**) was performed but AR target genes (FKBP5 and PSA) along with CTGF and ZEB1 were tested. (**C**) As previously reported [9], J54 results in the degradation of full-length YAP1 and the accumulation of a cleavage product (Cl-YAP1), through a time and dose-dependent mechanism due to loss of Y407 phosphorylation and resultant destabilization. (**D**) Accumulation of E-CAD, a marker of EMT reversal, was seen in YAP-WT-overexpressing cells treated with J54. This also resulted in the degradation of GFP-YAP1-WT, but not the GFP-YAP-Y407F, which was instead constitutively degraded. Two-way ANOVA statistical tests were performed within each row, (column effect), and where differences among groups were present, they were found at *p* < 0.0001 (or less) level of significance.

In order to establish this, we determined the growth rate of the LNCaP-expressing Green Fluorescent Protein (GFP) only, GFP-YAP1-WT, or Y407F mutant using the Incucyte system. It was immediately noticeable that the GFP-YAP1-WT-expressing cells were morphologically different from the Y407F mutant. The cells were round rather than spindly and tended to grow in loose clusters and often on top of each other (Figure 1D). The general impression was that they had a more mesenchymal appearance, which was not seen in the control GFP-LNCaP cells or in the Y407F mutant (Figure 1D). Moreover, only the YAP1-WT-expressing cells became androgen-independent (AI) for growth (could grow well with charcoal-stripped-serum, i.e., CSS - Figure 3, middle panel). Note that while the GFP-YAP1-WT-expressing cells and the Y407F mutants had similar doubling times, the former tended to grow in overlapping cells clusters and with time did not occupy as much surface area as the Y407F cells (Phase Object confluence, the parameter measured by the Incucyte). However, this did not change the result that their proliferation rate was essentially the same in the medium with FCS or CSS. Interestingly we observed from a live cell microscopy of LNCaP-GFP-YAP1 WT and Y407F cells that while GFP-YAP1-WT was nuclear, the Y407F mutant was predominantly partitioned into the cytoplasm (Figure S3). The most direct interpretation of this different nuclear/cytoplasmic distribution is that phosphorylation of Y407 results in increased interaction with YAP transcriptional partners, resulting in nuclear retention and increased activity, which is obviated with the Y407F mutant. It was previously reported that YAP1, via interaction with the AR, can convert LNCaP cells to AI [5], but clearly the Y407F mutant lacks this capacity, possibly having reduced transactivation. To establish this, we monitored the expression of a panel of important transcripts that reflect the activity of YAP1/TEAD and the AR in the three LNCaP derivatives. As representative of AR-driven transcripts, we determined FKBP5 and Kallikrein 3 (PSA) expression (Figure 4). 

### 3.4. Analysis of Transcriptomic Changes in LNCaP Cells Overexpressing GFP-YAP

When we analyzed the YAP/TEAD-dependent transcripts, we confirmed that in the YAP-expressing cells, Zeb1 and Twist1 (referred as Twist), two key transcriptional repressors of E-cadherin and downstream targets of YAP/TEAD, were oppositely regulated in the LNCaP-YAP 1-WT and Y407F-expressing cells. However, E-cadherin (E-CAD) was strongly reduced in the YAP1-WT cells, and also in the Y40F mutant (Figure 4) possibly through a more complex reciprocal regulation by Twist and YAP1 [23,24]. In fact, the reciprocally regulated Twist target gene N-cadherin (N-CAD) was suppressed only in the Y407F-expressing cells. Downregulation of Twist and N-cadherin was not unexpected, as YAP1 is known to regulate these mesenchymal markers. There is also significant cooperation between Snail and Twist in regulation of Zeb1 expression [25], a third key regulator of E-Cadherin expression and the epithelial to mesenchymal transition (EMT). However, the most important observation was that J54 (the inhibitor of the TLK1>Nek1>YAP pathway) completely reversed the altered transcription phenotype by YAP1 overexpression. In fact, it resulted in a dramatic increase in E-Cadherin re-expression in both YAP1-expressing lines and was significantly higher in WT, a clear marker of MET (mesenchymal to epithelial transition), and in the same cells, it also resulted in loss of N-cadherin.

Notably, in the Y407F mutant cells there was an even higher expression of Twist than in the untreated cells or cells expressing GFP-YAP1-WT (Figure 4, with J54). Since NEK1 was unable to phosphorylate the Y407 site in the Y40F mutant, the effect of J54 in these cells requires an alternative explanation, and we suggest that this may be due to NEK1-mediated phosphorylation of one of its other five specificity sites on YAP1 [9]. Regardless, this remarkable increase in E-Cadherin expression after J54 addition in the lines overexpressing GFP-YAP1, even considering that parental LNCaP cells have good expression of this transcript [26], is clearly indicative of a reversal of their phenotype toward the MET transition and suggests less motile and invasive properties. The increase in E-Cadherin protein expression in J54-treated cells overexpressing GFP-YAP1 was also evident (Figure 4D), although not quite as impressive as the mRNA elevation. The strong increase in E-Cadherin expression is best correlated with the loss of Zeb1 expression with J54, that can be consequent to the loss of YAP1 protein (Figure 4C, D-J54 (GFP-YAP1 or) YAP1 dose-dependent degradation), which is a regulator of Zeb1 [27].

Likewise, untreated GFP-YAP1-WT-overexpressing cells, more so than the Y407F mutants, display a more mesenchymal transcriptome and phenotypic morphology. Perhaps this is due to the parallel reduction in N-Cadherin expression in Y407F, resulting in what is known as a “hybrid” EMT phenotype [28], and confirming that YAP1 overexpression is key to these gene expression alterations. Another classic marker of YAP/TEAD activity, CTGF, was poorly expressed in LNCaP cells and was not different between the three lines, or only a 2-fold increase in the Y407F mutant. However, the addition on J54 almost completely abolished CTGF expression in all both WT and Y407F lines. 

We also tested the expression of two AR target genes, as YAP1 is known to be a transcriptional co-regulator and binding partner of AR, enhancing its activity (3) [4,5,29] Overexpression of GFP-YAP1-WT resulted in a dramatic (25-fold) increased expression of FKBP5 (Figure 4B), in contrast to only 2-fold in the Y407F mutant, which demonstrated its weaker transcriptional enhancement of the AR program-FKBP5 is a strongly AR-dependent gene as clearly established in LNCaP cells [30]. This would be consistent with the previous observation that only the YAP1-WT-expressing LNCaP cells were androgen-independent for growth (could grow in a CSS medium), suggesting an autonomous capacity to integrate AR transcriptional signals in a medium largely depleted of steroids. To our surprise, PSA was not similarly increased, and its expression was slightly reduced in the YAP1-WT-expressing LNCaP cells. The regulation of PSA by the YAP1/AR co-regulation may be more complex than seen from a previous report [5]. Importantly, YAP1 integrated AR and TEAD-dependent transcriptional pathways, leading to AI growth. EMT differentiation of LNCaP cells expressing GFP-YAP1-WT visibly and transcriptionally occurred, but much less so for the Y407F mutant (Figure 4A,B).

Even more dramatically, J54, the inhibitor of the TLK1>NEK1>YAP1 axis, resulted in the reversal of all EMT and AR transcriptional changes. This was showcased by the dramatic loss of FKBP5 expression at the mRNA level, and to a lesser extent in the protein level, due to the short duration of the treatment (Figure 4B,D). It is important to restate that treatment of LNCaP cells with J54 resulted in a dose- and time-dependent degradation of (GFP tagged or) YAP1 (Figure 4C,D) [9] and concomitantly, a reversal of the overexpression of FKBP5 that represents a marker of the AI program implemented by AR and YAP1-WT integration. Therefore, it is conceivable that treatment of a sub-population of CRPC patients wherein their PCa cells rely partly on YAP1 upregulation and/or Y407 phosphorylation may benefit from a concomitant antiandrogen and J54 combination. Similar decay of exogenous GFP-YAP1-WT (as well as the endogenous YAP1) was observed with J54 (Figure 4D), but not with the Y407F mutant, in which the stabilizing phospho-residue was of course mutated. The GFP-YAP-Y407F mutant was, instead, largely degraded constitutively (Figure 4D) as previously shown.

### 3.5. YAP1 Expression Is NEK1-Dependent in a Castrated TRAMP Mouse Model

**Figure 5:**
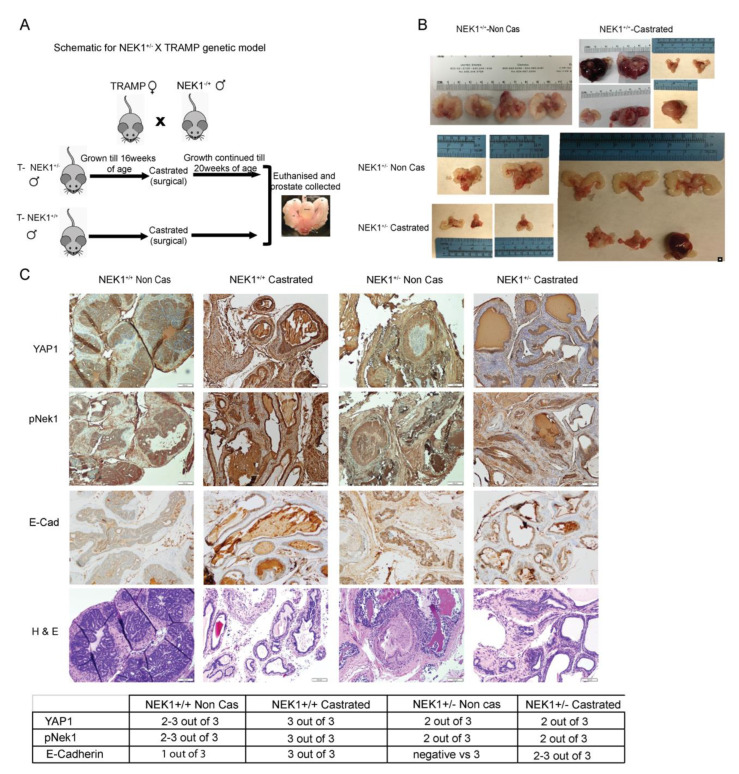
YAP1 expression was NEK1-dependent in a castrated TRAMP mouse model. (**A**) Schematic of the generation of TRAMP-*NEK1^+/+^* and TRAMP-*NEK1^+/−^* mouse models and an experiment timeline for PCa recurrence shown. (**B**) Prostate tissue morphology demonstrated from four different categories of mouse samples TRAMP-*NEK1^+/+^* (Non-castrated, Non-Cas), TRAMP-*NEK1^+/+^* (Castrated), TRAMP-*NEK1^+/^*^−^ (Non-castrated, Non-Cas) and TRAMP-*NEK1^+/−^* (Castrated). (**C**) Serial sections of the prostate tumor from TRAMP-*NEK1^+/+^* or TRAMP-*NEK1^+/−^* (Non-Cas or Castrated) mouse stained with anti-YAP1, anti-pNek1, anti- E-Cad (epithelial cell marker) and hematoxylin & eosin (H&E) as indicated. IHC score shown in table below. Scale bar 100 μm.

Since YAP1 is a proto-oncogenic factor, and our results so far indicate that YAP1 stability is NEK1-dependent, we wanted to test if the NEK1–YAP1 axis recapitulated in a mouse model. As YAP1 serves as the co-activator of AR in PCa model [4,5,29], we generated a NEK1KO (NEK1^+/−^) -TRAMP genetic mouse model (Figure 5A). These mice developed PIN and hyperplasia by 16 weeks of age, which progressed to prostate adenocarcinomas (PRAD) if the mice were left intact. However, if castrated and sacrificed at 20 weeks of age, when this in parental TRAMP marked the CRPC clinical stage, and there was a profound difference in PCa progression. We found that the NEK1^+/+^ parental TRAMP mouse developed PRAD (Figure 5B) even after castration, whereas the NEK1^+/−^-TRAMP mouse castrated at 12 weeks did not progress from PIN and hyperplasia to overt PRAD and CRPC. This suggests that NEK1 clearly plays a role in PCa progression.

Our quantified IHC data from prostate tissues showed that YAP1 expression was increased in castrated NEK1 parental-TRAMP mouse tissues, but significantly reduced in NEK1^+/−^-TRAMP castrated mouse prostate tissues (Figure 5C). A parallel H&E staining showed the hyperplasia and PIN lesions, and not PRAD, in an example of a castrated NEK1^+/−^-TRAMP animal. We also determined the expression of N-cadherin (N-Cad), which is known to be partly under the regulation of YAP1 and to contribute to the invasive capacity of PRAD cells, in intact vs. castrated animals, and in NEK1^+/+^ compared to NEK^1+/−^. Strikingly, intact NEK1^+/+^ mice sacrificed at 20 weeks demonstrated high N-Cad expression in the luminal PCa, and a much lower but still evident expression after castration, more so in the stroma as previously reported [26]. Importantly E-Cad expression increased in NEK1 parental-TRAMP mouse post castration and correlated with YAP1 expression but was less intense stained in NEK1^+/−^- TRAMP prostate tissue after castration (Figure 5C, IHC scores in lower panel). In stark contrast, in NEK1 haploinsufficient mice staining for N-Cad was negative (Figure S4), whether castrated or not, and regardless of evidence of overt PRAD lesions (intact mice) or just PIN (castrated mice). This supports the idea that the NEK1-mediated phosphorylation and stabilization of YAP1 may be critically important for EMT progression whether the animals are castrated or not, and hence, for metastatic progression. Indeed, we previously reported the effects of castration and concomitant inhibition of TLK1 with THD (precursor of J54) on the phosphorylation of NEK-T141 and on proliferative (Ki67) or apoptotic markers (Cl-Cas3 and Cl-PARP) in the TRAMP model [31].

### 3.6. Analysis of YAP1 and pYAP1-Y407 in Pca and Correlation with Gleason Score (GS)

**Figure 6:**
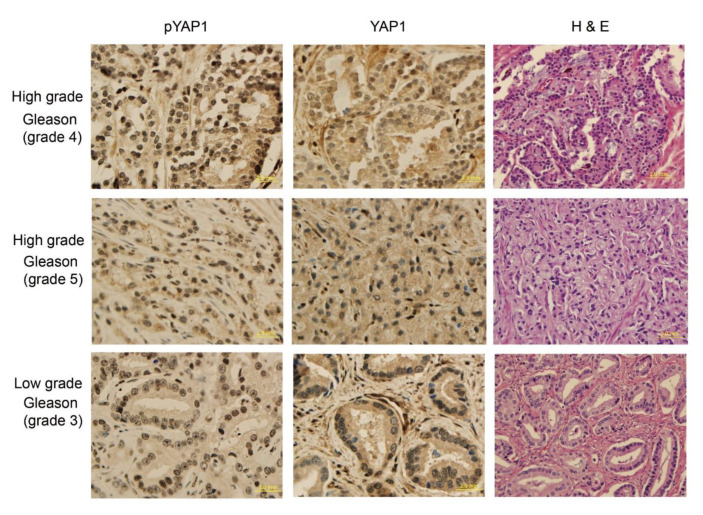
pYAP1(Y407) expression in nucleus of human PCa cells correlated strongly with gleason score increase. Human prostate tissues were stained for pYAP1(Y407) custom generated antibody and YAP1 (40×) and H&E (20×) as indicated. Different Gleason grades (high or low) shown. Scale bar 20 μm.

To determine if the expression of pYAP1 is more crucial in human Pca to detect the stage of cancer, we tested a group of Pca biopsies of different Gleason grades to study if the phosphorylation of YAP1-Y407 could mark a specific stage of cancer progression, particularly where YAP1 nuclear localization may indicate its co-transcriptional role as opposed to its cytoplasmic indolent function [32]. It is well-established that the nuclear/cytoplasmic shuttling is regulated by the phosphorylation of YAP1 by different kinases [33]. In Figure 6, we show a representative selection of PCa biopsies with increased GS, after IHC with either panYAP1 or pYAP1-Y407 antisera. It was immediately apparent that the signal distribution was not equivalent. The nuclear signal of pYAP1-Y407 was seen for GS3 to high GS5 biopsies and showed a strong correlation with an increase in grade. The panYAP1 signal was mostly cytoplasmic, and only increased in the nuclei of high grade (GS5) sections. Importantly, the nuclear staining of pYAP1-Y407 was seen even in highly differentiated GS3 ductules, whereas YAP1 staining was weak and only cytoplasmic for panYAP1. In fact, we observed that staining intensity for YAP1 was not much different for GS3 PCa than it was for “normal” hyperplastic glands sections. Therefore, it is tempting to suggest that staining for pYAP1-Y407 could be a useful marker for potentially distinguishing between BPH and low grade PCa sections.

## 4. Discussion

YAP1/TAZ (60% identical) are the main effectors of the Hippo signaling pathway, which is involved in regulating organ size through controlling multiple cellular functions, including cell proliferation and apoptosis. The Hippo pathway responds to a variety of cellular cues, including cell–cell contact, mechanotransduction, and apico-basal polarity [1,2,3]. When the Hippo signaling is activated, kinases MST1/2 and LATS1/2 phosphorylate and inactivate YAP1 and TAZ. YAP1 and TAZ are transcriptional co-activators but lack DNA binding activity. Upon phosphorylation by MST and LATS kinases, they are sequestered in the cytoplasm, ubiquitylated by the β-TrCP ubiquitin ligase, and marked for degradation by the proteasome. YAP1/TAZ are usually inhibited by the cell–cell contact in normal tissues [1,2,3].

Specifically, it should be noted that YAP1 is a generally unstable protein whose turnover rate is strongly regulated by multiple stabilizing [18] or de-stabilizing phosphorylation events controlled by multiple kinases (see [2,3,7] for some reviews). The best-known destabilizing event is the large tumor suppressor 1 and 2 (LATS1/2), the core kinases of the Hippo signaling pathway that can phosphorylate YAP1 on Ser127, which creates a binding site for the 14-3-3 protein. The binding of 14-3-3 to pYAP1-S127 leads to its cytoplasmic sequestration [34,35]. Sequential phosphorylation by LATS1/2 on YAP1 Ser397 primes it for further phosphorylation by Casein kinase 1 (CK1δ/ε) on Ser400 and Ser403, which creates a phosphodegron motif in the transcriptional activation domain (TAD) for β-TrCP/SCF E3 ubiquitin ligase-mediated proteasomal degradation [3]. The phosphorylation of YAP1 by NEK1 on Y407 (Figure 1A), which is located in the TAD, was a new finding by our lab and immediately correlated with its stabilization, since the pharmacologic inhibition of the TLK1>NEK1 nexus with THD or J54 resulted in a dose and time-dependent degradation of YAP1 (Figure 4B and [9]). Thus, it is notable that the over-activation of YAP1 can be directly suppressed via inhibition of the TLK1>NEK1 activation loop.

Over-activation of YAP1/TAZ through the aberrant regulation of Hippo kinases has been noted in many types of tumors and is associated with the acquisition of malignant traits, including resistance to anticancer therapies, maintenance of cancer stem cells, distant metastasis [1,2,3], and in prostates, AI adenocarcinoma progression [5,6]. When the Hippo core kinases are “off,” YAP1/TAZ translocate into the nucleus, bind to TEAD1-4, and activate the transcription of downstream target genes, leading to multiple oncogenic activities, including loss of contact inhibition, cell proliferation, epithelial–mesenchymal transition, and resistance to apoptosis.

In PCa, YAP1 has been identified as a binding partner of AR and co-localized with AR in an androgen-dependent manner and in an AI manner in CRPC [5]. YAP1 was also found to be upregulated in LNCaP-C4-2B cells and, when expressed ectopically in LNCaP, activates AR signaling and confers castration resistance, motility, and invasion (rev. in [4]). Knockdown of YAP1 greatly reduces the rates of migration and invasion of LNCaP, and YAP1-activated AR signaling was sufficient to promote LNCaP cells from an AS state to an AI in vitro and to castration resistance in vivo [5]. It also was recently determined that ERG (and the common *TMPRSS2-ERG* rearrangement) activates the transcriptional program regulated by YAP1, and that prostate-specific activation of either ERG or YAP1 in mice induces similar transcriptional changes and results in age-related prostate tumors [29]. However, the activators of the Hippo/YAP1 in PCa remain unclear.

We propose that TLKs have a role in this via activation and induced stabilization or nuclear relocalization via phosphorylation by NEK1 and in fact, we observed that GFP-YAP1-WT was mostly nuclear whereas the Y40F mutant was largely excluded from nuclei, where it performs its co-activator function (Figure S4). However, direct evidence of increased participation of pYAP-Y407 in transcriptional complexes with TEAD, AR, or *TMPRSS2-ERG* via coIP or ChIP at target loci remains the next chapter of this work. Our bioinformatics analysis suggested a link between NEK1 and YAP1 in different cancers [9]; the YAP1 protein is also abundant in high grade PCa tumors despite the progressive downregulation of YAP1 mRNA expression [9]. We propose that the signaling of TLK1 > NEK1-mediated YAP1 phosphorylation contributes to its stabilization, and found a correlation between increased pNEK1(T141) in relation to the GS and YAP1 protein expression [9,31], whereas its mRNA decreased, consistent with our model of post-transcriptional protein stabilization.

In this work we consolidated the critical importance of the Y407 phosphorylation for the stability and co-activator function of YAP1, as demonstrated most vividly from the fact that overexpression of GFP-YAP1-WT can transform LNCaP cell to a more “mesenchymal” and AI phenotype. However, the Y407F mutant, even when expressed at a higher level, could not fully convert LNCaP cell to AI and retained a normal spindly morphology. In addition, it was clear that the YAP1-Y407F mutant had high turn-over rates and was highly degraded and only expressed as full-length if continuously synthesized (Figure 1C,E).

We also validated that NEK1 was the principal (if not the only kinase) that phosphorylated Y407 by showing that depleting NEK1 with siRNA resulted in significant loss of the pYAP-Y407 signal. We further demonstrated that the YAP1-WT showed generally better transcriptional outputs in each pathway we investigated, such as the androgen-responsive genes, than the Y407F mutant. Perhaps paradoxically, because of the same reason, Hek293 cells expressing the Y407F mutant displayed better survival from MMC treatment than YAP1-WT, most likely because of a reduced interaction with p73 that implements the pro-apoptotic program [20,36]. Importantly, J54 can mediate the loss of YAP1 expression in LNCaP cells and presumably their derivates (Figure 4B) and was able to reverse the EMT phenotype of YAP1 overexpression—and, for instance, restore E-cadherin expression and FKBP5 back to the normal level (Figure 4). Whether this is accompanied by a reversal of the AI growth and MET phenotype of the YAP1-WT overexpressing LNCaP cells remains to be investigated. However, importantly, it was also shown recently that YAP1 regulates cell mechanics by controlling focal adhesion assembly [37] that is held to be critical for promoting motility and metastases, in addition to our new proposed role in AI progression.

In an earlier study with LNCaP xenografts, we reported that in animals treated with Bicalutamide (antiandrogen) in combination with J54, the tumors fail to recover AI growth after a lag (the normal adaptation for LNCaP sub-cutaneous- xenografts) and instead progressively shrink [13]. Therefore, it is tempting to propose that concomitant treatment with J54 and ADT may result in better cancer control, if not a real reversal, of mCRPC progression. In this regard, it was critical to establish in actual PCa biopsies the pattern of YAP1 and pYAP1-Y407 expression in relation to disease grade (GS) and to obtain an estimate of the proportion of cases that may be assumed to progress more rapidly to AI if their cancer is partly driven by AR/YAP1 integration. 

We also set about to determine if pYAP1-Y407 could be used as a marker to study PCa of progressively greater stage. This was studied by selecting a collection of PCa biopsies with Gleason Score—GS3 (low) to GS5 (high) via IHC staining and using the pYAP1-Y407 antiserum in comparison to a panYAP1 serum, which revealed that the distribution of pYAP1-Y407 was predominantly nuclear, suggesting that it is a “active” form of the protein capable of AR or TEAD transactivation. In contrast, panYAP1 signal was mostly cytoplasmic (the indolent form) in lower GS specimens. 

In conclusion, we found a novel pathway of activation of YAP1, rather than the canonical Hippo regulation, that relies on the sequential activation of the kinases TLK1 and NEK1 and led to the stabilization and activation of YAP1 by the phosphorylation of Y407. Inhibiting this kinase cascade with J54 can suppre ss some the transcriptional changes that are seen when YAP1-WT is overexpressed, at least in the LNCaP model.

## Data Availability

Description of all data and materials can be found in the referenced article. No additional data have been withheld from the public.

## Supplementary Materials

The following supporting information can be downloaded at: https://www.mdpi.com/article/10.3390/biomedicines11030734/s1. Figure S1: pYAP1 is decreased with depletion of NEK1. Hek293 cells treated with siNEK1 and immunoblotted for pYAP1-Y407; Figure S2: Treatment with MMC leads to accumulation of pYAP-Y407, whereas depletion of NEK1 results in its reduction. MMC results in activation and increased expression of NEK1 leading to pY407 increase, whereas depletion of NEK1 with siRNA [27] reduces it. Note that NEK1 displays various protein isoforms migrating between 120 and180 kDa [38]; Figure S3: GFP-YAP1 WT is nuclear and GFP-YAP1-Y407F is cytoplasmic in LNCaP cells. White arrows indicate GFP-YAP1 intracellular partitioning. Scale bar 100 μm; Figure S4: N-Cad staining of GEMM castrated and intact. Scale bar 100 μm. Inset shown at 400×.

## Abbreviations

AD/I	Androgen-dependent or -independent
ADT	Androgen Deprivation Therapy
AR	Androgen Receptor
ATR	Ataxia Telangiectasia Related
BAX	Bcl2 Associate Apoptosis protein X
CSS	Charcoal-Stripped Serum
EMT/MET	Epithelial to Mesenchymal Transition and vice versa
FKBP5	FK506 binding protein 5 (an immunophilin)
GFP	Green Fluorescent Protein
GS	Gleason Score
H&E	Hematoxylin-Eosin stain
mCRPC	Metastatic Castrate Resistant Prostate Cancer
MMC	Mitomycin C
NEK1	NIMA Related Kinase 1
NEPC	Neuroendocrine Prostate Cancer
PCa	Prostate Cancer
PIN	Prostate Intraepithelial Neoplasia
PRAD	Prostate Adenocarcinoma
TAD	Transcription Activation Domain
TRAMP mice	Transgenic Adenocarcinoma of the Mouse Prostate
