# Supplementary material for: NEK1-Mediated Phosphorylation of YAP1 Is Key to Prostate Cancer Progression
Source: Biomedicines. 2023 Feb 28;11(3):734. doi: 10.3390/biomedicines11030734 (PMC10045622; doi:10.3390/biomedicines11030734)
Supplement: Supplementary file 1 [file biomedicines-11-00734-s001.zip › biomedicines-2201525-supplementary.pdf]

Figure S1: pYAP1 is decreased with depletion of NEK1

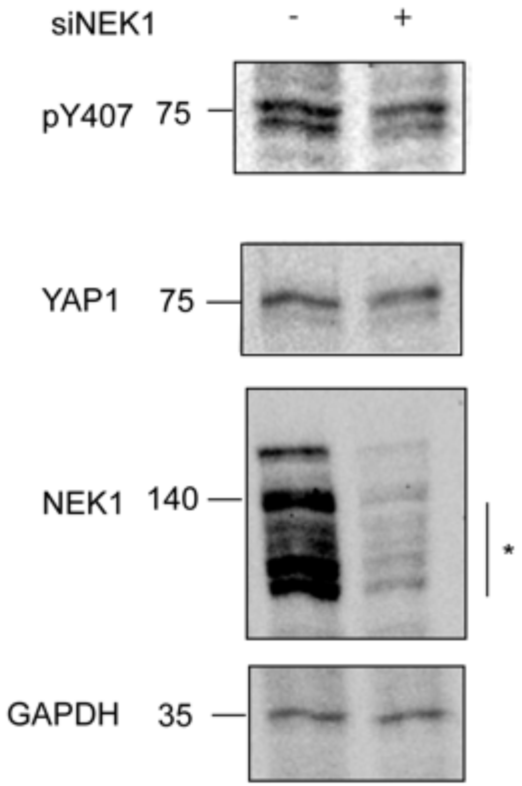

Figure S2: pYAP1 expression in Hek293 parental cells post MMC induction increases by 50%

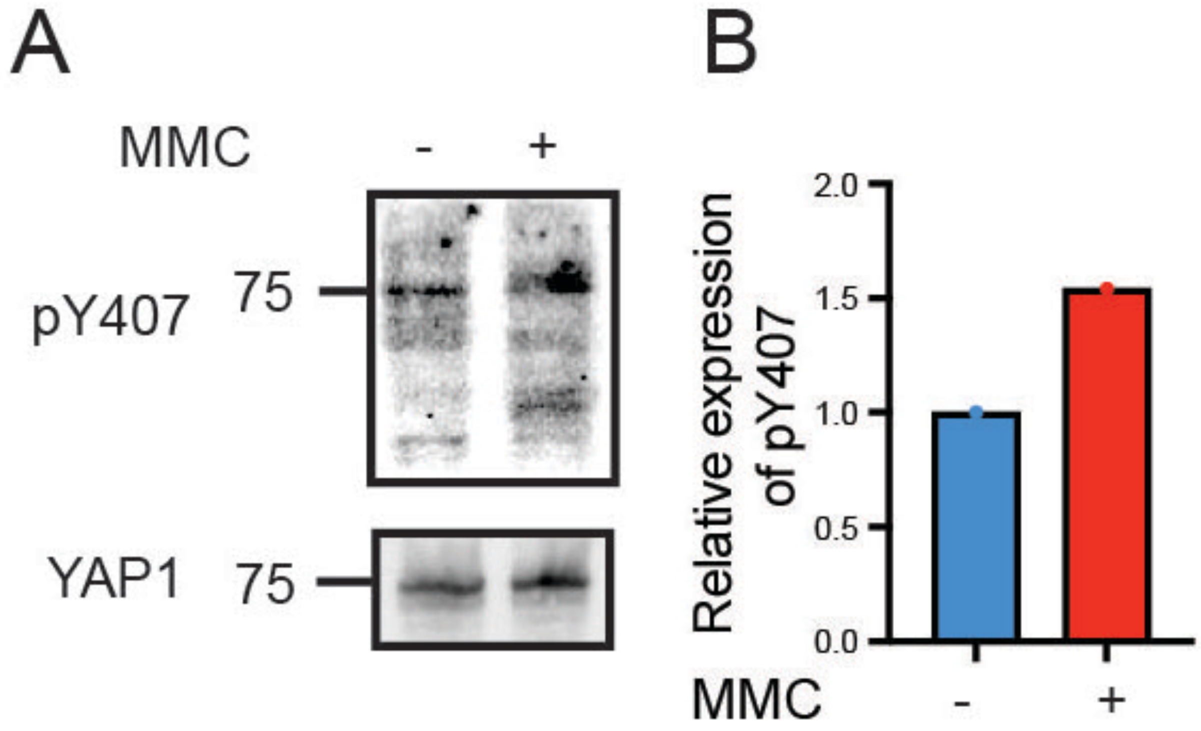

Figure S3: GFP-YAP1 WT is nuclear and GFP-YAP1-Y407F is cytoplasmic in LNCaP cells.

Data acquired from live LNCaP-GFP-YAP1-WT and Y407F cells in Incucyte objective at 10X. Phase contrast images shown in panel A and Fluorescence images in panel B. Scale bar 100μm.

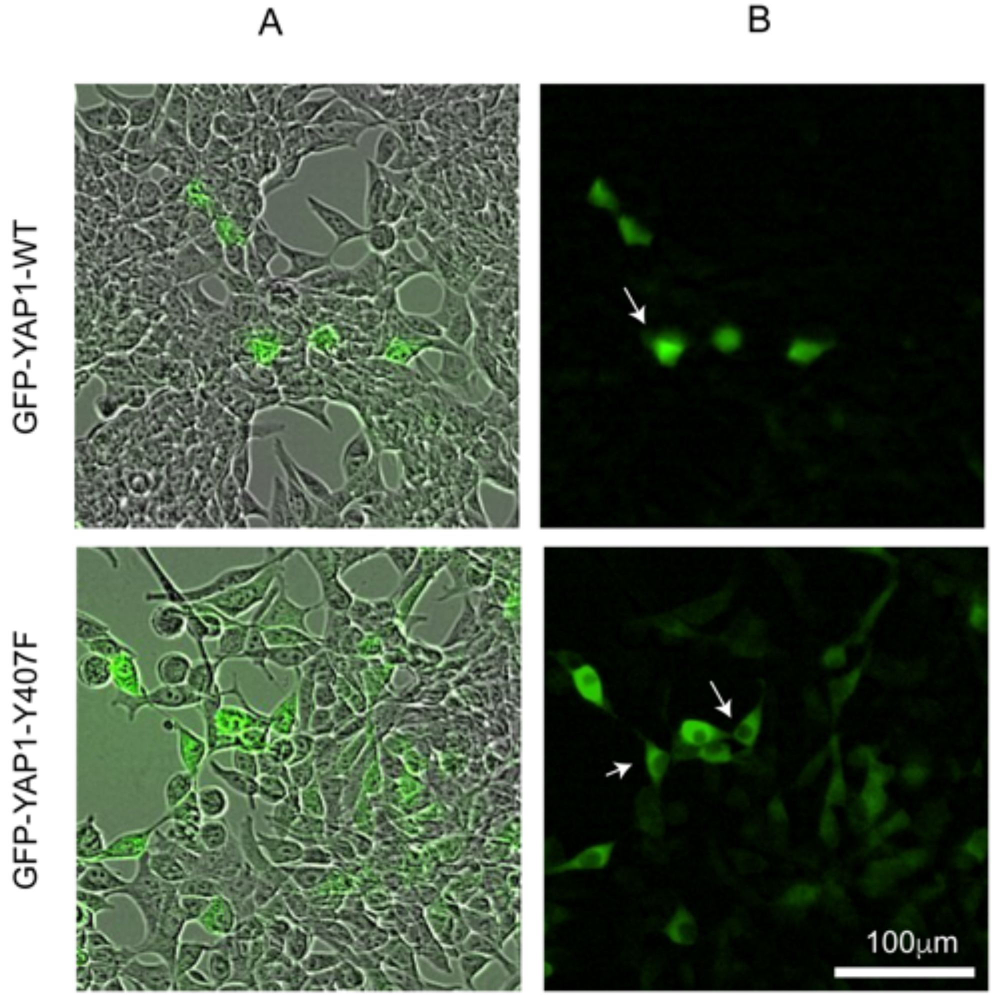

Figure S4: N-Cad probed in TRAMP-*NEK1*$^{+/+}$ (Non-castrated, Non-Cas), TRAMP-*NEK1*$^{+/+}$ (Castrated), TRAMP-*NEK1*$^{+/-}$ (Non-castrated, Non-Cas) and TRAMP-*NEK1*$^{+/-}$ (Castrated). Scale bar 100μm . Inset shown at 400X.

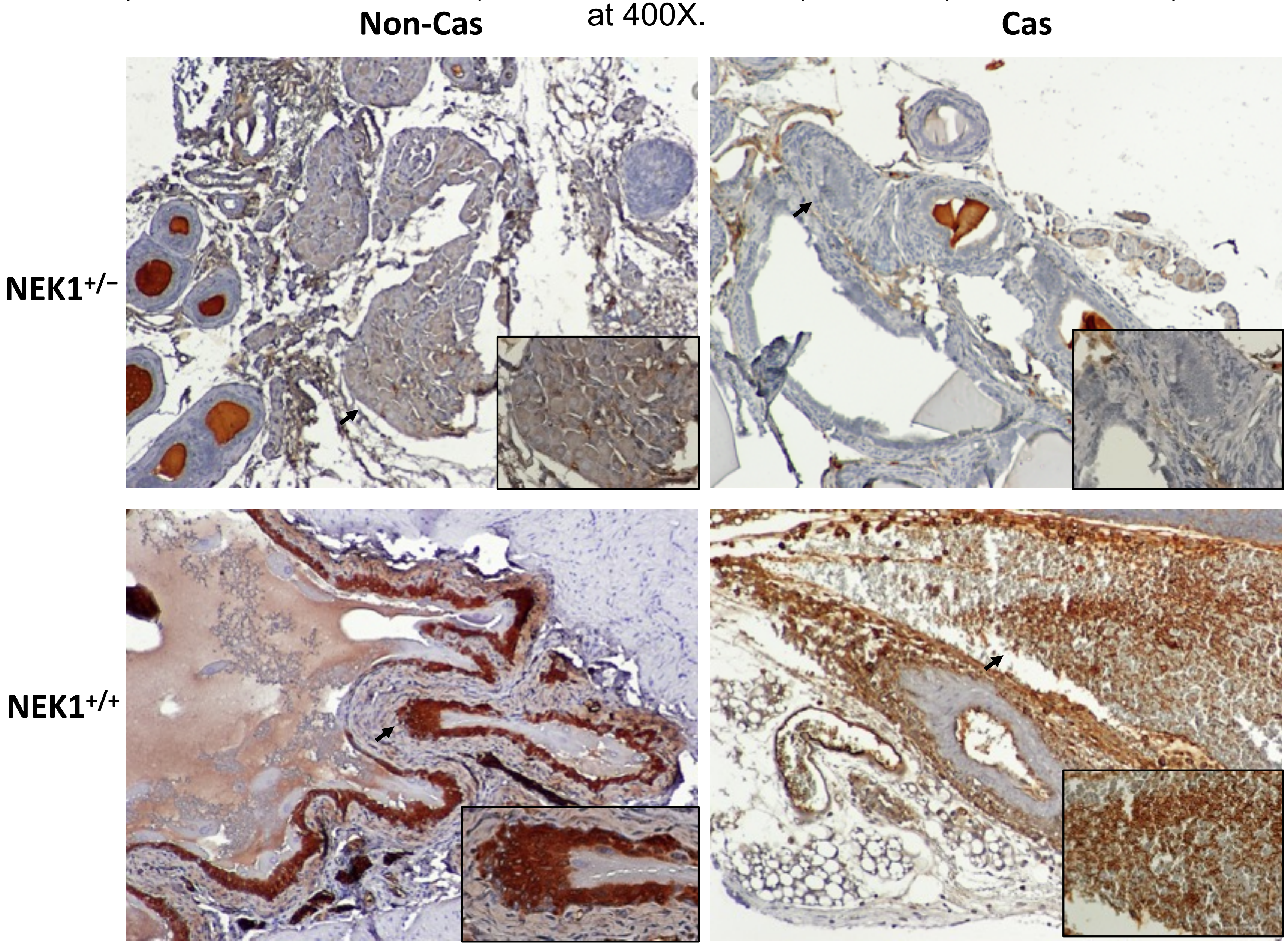

Arrowed portions have shown in insets
